# Antiangiogenic Potential of an Olive Oil Extract: Insights from a Proteomic Study

**DOI:** 10.1021/acs.jafc.3c08851

**Published:** 2024-05-29

**Authors:** Ana Dácil Marrero, Casimiro Cárdenas, Laura Castilla, Juan Ortega-Vidal, Ana R. Quesada, Beatriz Martínez-Poveda, Miguel Ángel Medina

**Affiliations:** † Departamento de Biología Molecular y Bioquímica, Facultad de Ciencias, Andalucía Tech, Universidad de Málaga, E-29071 Málaga, Spain; ‡ Instituto de Investigación Biomédica y Plataforma en Nanomedicina-IBIMA Plataforma BIONAND (Biomedical Research Institute of Málaga), E-29071 Málaga, Spain; § CIBER de Enfermedades Raras (CIBERER), Instituto de Salud Carlos III, E-28029 Madrid, Spain; ∥ Servicios Centrales de Apoyo a la Investigación (SCAI), Universidad de Málaga, E-29071 Málaga, Spain; ⊥ Departamento de Química Inorgánica y Orgánica, Campus de Excelencia Internacional Agroalimentaria ceiA3, Universidad de Jaén, Jaén E- 23071, Spain; # CIBER de Enfermedades Cardiovasculares (CIBERCV), Instituto de Salud Carlos III, E-28029 Madrid, Spain

**Keywords:** virgin olive oil, mediterranean diet, natural extract, angiogenesis, chemoprevention, functional food

## Abstract

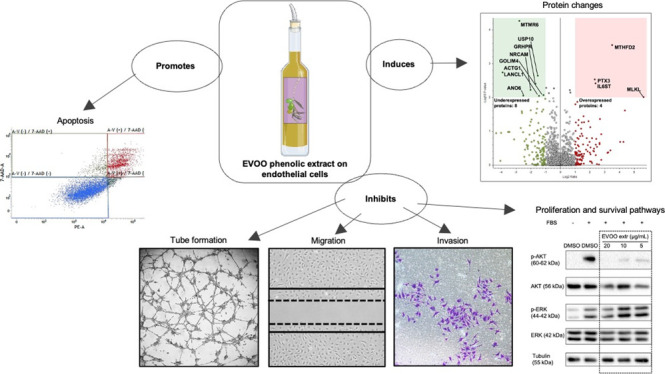

Extra virgin olive oil (EVOO), a staple of the Mediterranean diet, is rich in phenolic compounds recognized for their potent bioactive effects, including anticancer and anti-inflammatory properties. However, its effects on vascular health remain relatively unexplored. In this study, we examined the impact of a “picual” EVOO extract from Jaén, Spain, on endothelial cells. Proteomic analysis revealed the modulation of angiogenesis-related processes. In subsequent in vitro experiments, the EVOO extract inhibited endothelial cell migration, adhesion, invasion, ECM degradation, and tube formation while inducing apoptosis. These results provide robust evidence of the extract’s antiangiogenic potential. Our findings highlight the potential of EVOO extracts in mitigating angiogenesis-related pathologies, such as cancer, macular degeneration, and diabetic retinopathy.

## 1 Introduction

Extra virgin olive oil (EVOO) is used as the main source of fat in Mediterranean dietary patterns. EVOOs are distinguished by their extraction exclusively through mechanical or physical processes under specific thermal conditions that preserve the oil’s integrity, such as washing, decantation, centrifugation, or filtration.^1^ These premium oils are never subjected to solvent extraction, re-esterification, or mixing with other oils.^1^ This meticulous processing ensures the retention of their health-promoting properties, primarily attributed to their minor components—the phenolic compounds.^1−3^ According to numerous studies, these effects are attributed to key secoiridoid derivatives such as ligstroside, oleuropein, oleocanthal, and oleacein and simple phenols like tyrosol and hydroxytyrosol.^4,5^

Remarkably, the composition of phenolic molecules can vary significantly depending on factors such as olive cultivar, variety, geographic origin, and harvest season.^2,6^ The Picual variety, commonly found in Mediterranean households, boasts a higher total phenolic content and antioxidant capacity compared to others, primarily attributed to its rich secoiridoid content.^7^ However, even within the same variety, the phenolic composition of EVOOs can exhibit variability influenced by these factors.^6^

It is widely recognized that EVOO plays a fundamental role in the health benefits attributed to the Mediterranean diet,^8^ encompassing well-documented anti-inflammatory and antioxidant properties,^9−12^ neuroprotective effects,^13−15^ and anticancer potential.^16−19^ Furthermore, it has received some attention, albeit limited, for its antiangiogenic properties.^20,21^

In this comprehensive study, we delve into the examination of the phenolic fraction found in EVOOs and its potential impact on endothelial cells with a particular focus on its role in angiogenesis. To accomplish this, we employed a phenolic extract derived from EVOO sourced from the Picual variety in Jaén, Spain. Our investigation of an extract offers a compelling advantage, enabling us to obtain larger quantities of bioactive molecules compared with conventional dietary consumption, provided that the concentrations of these diverse compounds within the mixture are deemed safe. Furthermore, this approach allows us to meticulously isolate and assess the effects of the phenolic fraction independently from the other components of EVOO, such as fatty acids and more.

## 2 Materials and Methods

### 2.1 Reagents

Dulbecco’s modified Eagle's medium (DMEM) 1 and 4.5 g/L glucose, endothelial cell growth basal medium-2 (EBM-2), endothelial cell growth medium-2 BulletKit (EGM-2), penicillin/streptomycin, amphotericin B, and l-glutamine were supplied by Biowhittaker-Lonza (Walkersville, MD, USA). Fetal bovine serum (FBS) was supplied by Capricorn Scientific (Ebsdorfergrund, Germany). The general reagents used for the different assays were obtained from Sigma-Aldrich (Merck; Darmstadt, Germany). Caspase-Glo 3/7 Assay kit was purchased from Promega Biotech Ibérica (Madrid, Spain), Matrigel was from Corning (New York, NY, USA), and PE Annexin V Apoptosis Detection Kit I was provided by BD Biosciences (San Jose, CA, USA). The anti-PARP (ref #9542S), ERK1/2 (ref #4695S), phospho-ERK1/2 (Thr202/Tyr204) (ref #4370S), AKT (ref #9272S), phospho-AKT (Ser473) (ref #9271S), CNN2-CTGF (ref #86641), α-tubulin (ref #3873S), and GAPDH (ref #2118L) antibodies were purchased from Cell Signaling (Danvers, MA, USA). EFEMP1 antibody (ref #TA503772) was purchased from Thermo Fisher Scientific (Waltham, MA, US). Solvents used for extraction of the olive oil sample, such as methanol (MeOH) and water, were purchased (analytical grade) from VWR Chemicals (Prolabo, Madrid, Spain) or produced by a Milli-Q water (1.8 MΩ) equipment (Merck, KGaA, Darmstadt, Germany), respectively.

### 2.2 Extraction of a Phenolic Extract from Extra Virgin Olive Oil (EVOO)

The olive oil sample used for extraction was purchased from the agricultural cooperative “San Ginés y San Isidro”, which produces olive oil with olives of the cultivar “Picual” (*Olea europaea* L. cv. Picual) (Sabiote, Jaén, Spain). The phenolic extract was obtained following a procedure based on that described by the International Olive Council (IOC, 2017): an olive oil sample (100 g) was extracted with a mixture of MeOH/H_2_O 8:2 (v/v) (3 × 100 mL); the upper phases and the oily phase were combined again and sonicated at room temperature for 15 min. The upper phases were decanted from the rest and were centrifuged at 3000 × *g* for 25 min with a centrifuge model Mixtasel-BL Selecta (JP Selecta, Barcelona, Spain). The resulting supernatant phases were combined and evaporated under vacuum at temperatures not higher than 40 °C. The dry extract (500 mg) was stored under argon at −20 °C until use.

For the in vitro assays, a 100 mg/mL stock solution of the EVOO extract was prepared in DMSO, from which dilutions were made as required.

### 2.3 Phenolic Profiling of the EVOO Extract

The qualitative profiling of the phenolic extract from EVOO was performed using the ultrahigh-performance liquid chromatography (UHPLC) system coupled to a high-resolution accurate mass spectrometer operating in a data-dependent acquisition mode as a nontarget approach.

#### 2.3.1 Ultrahigh-Performance Liquid Chromatography

The analysis was performed on a UHPLC system Easy nLC 1200 UHPLC (Thermo Fisher Scientific, Waltham, MA, US) coupled to a hybrid linear trap quadrupole Orbitrap Q-Exactive HF-X mass spectrometer (Thermo Fisher Scientific, Waltham, MA, US). Software versions used for the data acquisition and operation were Tune 2.9 and Xcalibur 4.1.31.9. The mobile phase consisted of phases A [0.1% formic acid (FA) in water] and B (0.1% FA in 80% acetonitrile). The chromatographic separation was performed on a 25 cm analytical column (PepMap RSLC C18, 2 μm, 100 A, 75 μm × 25 cm, Thermo Fisher Scientific, Waltham, MA, US) operated at 40 °C. The compounds were separated from the analytical column with a 60 min gradient ranging from 2 to 70% solvent B, followed by a 10 min gradient from 70 to 98% solvent B, and finally to 98% solvent B for 10 min at a constant flow rate of 300 nL/min.

#### 2.3.2 High-Resolution Mass Spectrometry

Ionization was performed with a nanoelectrospray ionization source (Thermo Scientific EASY-Spray) operating in positive mode. The LTQ Velos ESI Positive Ion Calibration Solution (Thermo Fisher Scientific, Waltham, MA, US) was used to externally calibrate the instrument prior to sample analysis, and an internal calibration was performed on the polysiloxane ion signal at *m*/*z* 445.120024 from ambient air.

MS1 scans were performed from *m*/*z* 100 to 1300 at a resolution of 120,000. Using a data-dependent acquisition mode, the 5 most intense precursor ions were isolated within a 2.0 *m*/*z* window and fragmented to obtain the corresponding MS/MS spectra. The fragment ions were generated in a higher energy collisional dissociation (HCD) cell and detected in an Orbitrap mass analyzer (Thermo Fisher Scientific, Waltham, MA, US) at a resolution of 30,000. The dynamic exclusion time for the selected ions was 10 s. Maximal ion accumulation time allowed in the MS and MS2 mode was 100 and 50 ms, respectively. Automatic gain control (AGC) was used to prevent overfilling of the ion trap and was set to 3 × 10^6^ and 10^5^ ions for a full MS and MS2 scan, respectively.

#### 2.3.3 Data Analysis

Data were processed using Compound Discoverer 3.3 software (Thermo Fisher Scientific, Waltham, MA, US). Compound annotation validation was set to a mass error of 5 ppm. Compound prediction based on the fragmentation pattern of the mass spectra of the compounds was performed using m/zCloud, ChemSpider, and the freely available Phenol-Explorer 3.6 databases.^22^

### 2.4 In Vitro Culture of Endothelial Cells

Human umbilical vein endothelial cells (HUVEC) were purchased from Lonza (Basel, Switzerland) and cultured in EGM-2 supplemented with a 1% penicillin/streptomycin solution until a maximum passage of 9. Bovine aortic endothelial cells (BAECs) were isolated as previously described^23,24^ and cultured in DMEM (1 g/L glucose), supplemented with a 1% penicillin/streptomycin solution, 2 mM l-glutamine, and 10% FBS, until a maximum passage of 20. Cervix adenocarcinoma (HeLa) and human gingival fibroblast (HGF-1) cell lines were maintained in DMEM (4.5 g/L glucose) supplemented with a 1% penicillin/streptomycin solution, 2 mM l-glutamine, and 10% FBS.

### 2.5 MTT Cell Survival Assay

Cell survival was determined in the presence of different compounds by the thiazolyl blue tetrazolium bromide (MTT) dye reduction assay as previously described by us.^24^ In 96-well plates, 4000 cells per well were seeded in the case of HUVEC and 2000 cells per well in the case of BAEC, HeLa, and HGF-1. IC_50_ values were calculated as the concentration of the compound that allows 50% of cell survival after 3 days of treatment, considering 100% the absorbance value of the control condition with dimethyl sulfoxide (DMSO). At least three independent replicates were performed for this assay. Concentrations in the range of the IC_50_ value for each compound in HUVEC and BAEC were used as the reference concentrations for the rest of the studies.

### 2.6 Proteomics Analysis

To investigate the effects of the EVOO extract on protein expression levels in HUVEC, a proteomics analysis was carried out.

#### 2.6.1 Cell Treatment and Protein Extraction

For proteomic analysis, HUVEC were incubated for 6 h in basal medium EBM-2 under two different experimental conditions: untreated controls or 10 μg/mL EVOO extract. After incubation, culture media were collected and centrifuged to remove cell debris, and supernatants were immediately frozen at −80 °C for further lyophilization. Meanwhile, cells were washed with ice-cold phosphate-buffered saline (PBS) and solubilized in 100 mM triethylammonium bicarbonate buffer (TEAB)-10% SDS containing Pierce universal nuclease (Thermo Fisher Scientific, Waltham, MA, US). Then, cells were sonicated and centrifuged at 16,000 *g* for 10 min at 4 °C, and the supernatant was carefully separated. After lyophilization, the culture media were reconstituted with 100 mM TEAB.

#### 2.6.2 In-Solution Tryptic Digestion and Peptide Extraction

The protein concentrations of the samples were determined by a fluorometric assay with the Qubit platform (Invitrogen, Carlsbad, CA, USA) and normalized to the same concentration (1 μg/μL). For reduction and alkylation, 5 μL of 200 mM Tris (2-carboxyethyl) phosphine was added, and the mixture was incubated at 55 °C for 1 h. Proteins were then alkylated with 60 mM iodoacetamide at room temperature for 30 min, protected from light. Afterward, samples were subjected to acetone precipitation to purify proteins by incubating with 6 volumes of ice-cold acetone at 20 °C for 4 h. Precipitated proteins were centrifuged at 8000 *g* for 10 min at 4 °C, and the pellet was redissolved in 100 μL of 50 mM TEAB (pH 8.5). Proteins were then digested by trypsin (Pierce trypsin protease, Thermo Scientific, Waltham, MA, US) at a ratio of 1:50 (trypsin:protein, w/w) by incubating overnight at 37 °C. Next, samples were dried in a SpeedVac (Thermo Fisher Scientific, Waltham, MA, USA) vacuum concentrator, redissolved in 50 μL of 0.1% FA, sonicated for 3 min, and centrifuged at 14,000 *g* for 5 min. Finally, samples were quantified again in a NanoDrop (Thermo Fisher Scientific, Waltham, MA, USA), and 0.1% FA was added to equalize all samples at an identical protein concentration before being transferred to the injection vial.

#### 2.6.3 Liquid Chromatography High-Resolution Mass Spectrometry (HPLC-MS)

Samples were injected onto an Easy nLC 1200 UHPLC system coupled to a hybrid linear trap quadrupole Orbitrap Q-Exactive HF-X mass spectrometer (Thermo Fisher Scientific, Waltham, MA, USA). Software versions used for the data acquisition and operation were Tune 2.9 and Xcalibur 4.1.31.9. UHPLC solvents were as follows: solvent A consisted of 0.1% FA in water, and solvent B consisted of 0.1% FA in 80% acetonitrile. From a thermostated autosampler, 2 μL that correspond to 100 ng of the peptide mixture was automatically loaded onto a trap column (Acclaim PepMap 100, 75 μm × 2 cm, C18, 3 μm, 100 A, Thermo Fisher Scientific, Waltham, MA, USA) at a flow rate of 20 μL/min and eluted onto a 50 cm analytical tube (PepMap RSLC C18, 2 μm, 100 A, 75 μm × 50 cm, Thermo Fisher Scientific, Waltham, MA, USA). The peptides were eluted from the analytical column with a 180 min gradient ranging from 2 to 20% solvent B, followed by a 30 min gradient from 20 to 35% solvent B and finally to 95% solvent B for 15 min before re-equilibration to 2% solvent B at a constant flow rate of 300 nL/min. The LTQ Velos ESI Positive Ion Calibration Solution (Thermo Fisher Scientific, Waltham, MA, USA) was used to externally calibrate the instrument prior to sample analysis, and an internal calibration was performed on the polysiloxane ion signal at *m*/*z* 445.120024 from ambient air. MS1 scans were performed from *m*/*z* 300 to 1750 at a resolution of 120,000. Using a data-dependent acquisition mode, the 20 most intense precursor ions of all precursor ions with +2 to +5 charge were isolated within a 1.2 *m*/*z* window and fragmented to obtain the corresponding MS/MS spectra. The fragment ions were generated in a HCD cell with a fixed first mass at 110 *m*/*z* and detected in an Orbitrap mass analyzer at a resolution of 30,000. The dynamic exclusion time for the selected ions was 30 s. Maximal ion accumulation time allowed in the MS and MS2 mode was 50 ms. AGC was used to prevent overfilling of the ion trap and was set to 3 × 106 and 105 ions for a full MS and MS2 scan, respectively.

#### 2.6.4 Data Analysis for Protein Identification

The acquired raw data were analyzed in the Proteome Discoverer 2.5 (Thermo Fisher Scientific, Waltham, MA, USA) platform with the SEQUEST HT engine using mass tolerances of 10 ppm and 0.02 Da for precursor and fragment ions, respectively. Two missed tryptic cleavage sites were allowed. Oxidation of methionine and N-terminal acetylation were set as variable modifications, while carbamidomethylation of cysteine residues was set as fixed modification. Peptide spectral matches (PSM) and consecutive protein assignments were validated using the Percolator algorithm^25^ based on a target-decoy approach using a reversed protein database as the decoy by imposing a strict cutoff of 1% false discovery rate (FDR). Peptide identifications were grouped into proteins according to the law of parsimony, and the results were filtered to contain only proteins with at least two unique peptide sequences.

#### 2.6.5 Label-Free Relative Quantification for Differential Expression Analysis

Label-free quantitation was implemented using the Minora feature of Proteome Discoverer 2.5 (Thermo Fisher Scientific, Waltham, MA, USA), setting the following parameters: maximum retention time alignment of 10 min with a minimum S/N of 5 for feature linking mapping. Abundances were based on the precursor intensities. Normalization was performed based on total peptide amount, and samples were scaled on all averages (for every protein and peptide, the average of all samples is 100). The normalized and scaled relative abundance of every protein was expressed as the mean ± standard deviation (SD) of three biological replicates. Protein abundance ratios were directly calculated from the grouped protein abundances. Abundance ratio ρ-values were calculated by ANOVA based on the abundances of individual proteins or peptides. The MS proteomics data have been deposited to the ProteomeXchange Consortium^26^ via the PRIDE partner repository with the data set identifier PXD045447.

### 2.7 Endothelial Tubular-Like Structure Formation on Matrigel

Tubular-like structure formation on Matrigel was assayed as previously described.^24^ Briefly, BAEC (5 × 10^4^ cells in FBS-free medium) were seeded onto a Matrigel layer in the presence of different doses of the EVOO extract for 4–5 h. A negative control (DMSO) and a positive control of the inhibition of tubular structures (2 μM staurosporine) were included. The number of formed tubular structures compared to the control was analyzed, and the minimum inhibitory concentrations (MIC) were determined. Those concentrations that inhibited the formation of closed tubular-like structures were considered positive in terms of complete inhibition of the process. A minimum of three independent replicates were used for this assay. After the incubation time, pictures were taken with a Nikon DSRi2 camera connected to a Nikon Eclipse Ti microscope (Nikon, Minato, Tokyo, Japan), and the tubular structures were analyzed using ImageJ software (NIH, National Institutes of Health of the United States)

### 2.8 Migration Assay (Wound-Healing Assay)

The migratory activity of BAEC was determined by the wound-healing assay as previously described.^24^ After creating the wound on confluent cultures, BAEC were incubated with DMSO or different doses of EVOO extract for 7 h to allow for migration. In each experimental condition, images of the scratched area at two time points (0 and 7 h) were captured using a Nikon DSRi2 camera connected to a Nikon Eclipse Ti microscope (Nikon, Minato, Tokyo, Japan). Subsequently, the cell-free area after 7 h of incubation was analyzed using ImageJ software (NIH, National Institutes of Health of the United States), normalizing it concerning the values at time zero.

### 2.9 Adhesion to the Fibronectin Assay

The endothelial capacity to adhere to fibronectin was evaluated. Subconfluent BAEC were incubated for 24 h in the presence or absence of different doses of the EVOO extract. Simultaneously, 24-well plates were treated with a 10 μg/mL fibronectin solution and blocked with a 3% BSA solution in PBS for an extra hour. Next, 9 × 10^3^ cells/mL suspensions were added to the fibronectin-treated-24-well plates. After 1 h of incubation, wells were gently washed three times, and cells that remained attached were photographed and counted.

### 2.10 Invasion Assay on Matrigel

The invasive potential of ECs was assayed with Matrigel-coated transwells as previously described in ref (27). BAEC were grown until subconfluency and were then incubated overnight in FBS-free media supplemented with 0.1% BSA. Next, cells were pretreated with DMSO or different doses of the EVOO extract for 15 min, and 5 × 10^4^ cells were then seeded on the transwells, facing media supplemented with 20% FBS. A negative control of invasion (without chemoattractant) was included. After overnight incubation, invading cells were fixed in 4% paraformaldehyde and stained with a 1% crystal violet solution in 2% ethanol. The percentage of cells in the bottom-facing surface of the transwell in the different conditions compared to the positive control (DMSO versus FBS-containing media) was quantified. Pictures were taken with a Nikon DSRi2 camera connected to a Nikon Eclipse Ti microscope (Nikon, Minato, Tokyo, Japan), and the tubular structures were analyzed using ImageJ software (NIH, National Institutes of Health of the United States).

### 2.11 Gelatin Zymography

Gelatin zymography of BAEC lysates and conditioned media was performed to assess the relative activity of matrix metalloproteinase 2 (MMP-2), largely expressed in BAEC, as previously described.^27^ BAEC were grown until subconfluency and then incubated with DMSO or different concentrations of the EVOO extract in FBS-free media supplemented with 0.1% BSA and containing 200 kallikrein inhibitor units (KIU) of aprotinin per mL. After 24 h of incubation, media and cell lysates were collected and processed. The gelatinolytic activity of the conditioned media was approached through densitometric analysis of the resulting bands. Gel signal was detected with an imaging system Chemidoc XRS (Bio-Rad, Hercules, CA, USA), and the densitometry analysis was performed using ImageJ software (NIH, National Institutes of Health of the United States).

### 2.12 Cell Cycle Analysis by Flow Cytometry

The cell cycle of BAEC was studied as previously reported.^27^ BAEC were cultured until subconfluency and then treated overnight with different doses of the EVOO extract. A negative control containing DMSO and a positive control of cell impairment consisting of 10 μM 2-methoxyestradiol (2-ME)^28,29^ were included. The percentages of cells in the G_0_/G_1_, S, and G_2_/M phases of the cycle, and the population in sub-G_1_ (fragmented DNA), were determined using a BD Biosciences FACS VERSE flow cytometer (Becton Dickinson, Franklin Lakes, NJ, USA). The resulting data was analyzed with the BD FACSuite program (Becton Dickinson).

### 2.13 Detection of Caspase 3 and 7 Activity

1.3 × 10^4^ were seeded in 96-well luminometry plates and were then incubated for 4 h to allow cell attachment. Next, cells were treated overnight with different doses of the EVOO extract. A negative control (DMSO) and a positive control of caspase 3 and 7 activity induction (10 μM 2-ME)^28,29^ were included in this assay. After treatment, caspase 3 and 7 activity was measured by adding the Caspase-Glo_3/7 reagent according to the manufacturer’s instructions, and luminescence was detected after 30 min with a GLOMAX 96 microplate luminometer (Promega Biotech Ibérica, Madrid, Spain).

### 2.14 Detection of Exposed Phosphatidylserine on the Cell Surface

The PE Annexin V Apoptosis Detection Kit I, from BD Biosciences Pharmingen (San Diego, CA, USA), was used to detect phosphatidylserine on the cellular surface of BAEC by flow cytometry according to the instructions of the manufacturer. BAEC were cultured until subconfluency and then incubated overnight with different doses of the EVOO extract. A negative control (DMSO) and a positive control of cell impairment (10 μM 2-ME)^28,29^ were included. The cytometer was a BD Biosciences FACS VERSE flow cytometer (Becton Dickinson), and the resulting data were analyzed with the BD FACSuite program (Becton Dickinson).

### 2.15 Western-Blot Protein Analysis

To validate the proteomic findings by Western blot, HUVEC were cultured until they reached confluency and were then incubated in basal media (without supplements) in the presence of DMSO or 10 μg/mL of EVOO extract for 6 h. Following the incubation period, the conditioned media were harvested, subjected to centrifugation at 1500 rpm for 5 min to remove debris, and stored at −80 °C. In parallel, cells were washed three times with ice-cold PBS and stored at −80 °C. For sample preparation, conditioned media were concentrated 50-fold through centrifugation using Amycon Ultra-4 tubes (Merck, Darmstadt, Germany) at 1500 rpm, and samples were then mixed with Laemmli sample buffer 2×. Regarding cell extracts, cells were directly lysed with Laemmli sample buffer 2×.

For apoptosis characterization assays, BAEC were incubated overnight in complete media with varying concentrations of the EVOO extract. DMSO served as the negative control (vehicle), while 10 μM 2-ME was used as the positive control. Following incubation, cells were lysed in 150 μL of RIPA buffer containing protease and phosphatase inhibitors. Protein concentration in the samples was determined using the DC Protein Assay (Bio-Rad, Hercules, CA, USA). Subsequently, 15–30 μg of total protein from each sample was subjected to SDS-PAGE denaturing electrophoresis.

To explore AKT and ERK 1/2 signaling pathways, BAEC were serum-starved for 24 h. After 22 h of serum starvation, cells were treated for an additional 2 h with varying concentrations of the EVOO extract prepared in fresh serum-free media. Following this, cells were stimulated with medium containing FBS for 10 min. A nonactivated control condition was also included. Subsequently, cells were processed as described in the previous paragraph.

Upon sample collection, samples were denaturalized and subjected to SDS-PAGE electrophoresis. In the case of protein result validation, equivalent amounts of conditioned media and cell extract samples were run simultaneously to allow for relative normalization. The separated proteins were transferred to nitrocellulose membranes. After the transfer, membranes were incubated overnight with antibodies against EFEMP1, CCN2, PARP1, AKT, ERK, and their phosphorylated forms, all diluted at 1:500–1000 in TBS-T with 5% BSA. Subsequently, membranes were exposed to secondary antibodies diluted at 1:5000 in a blocking buffer. Protein signals were detected by using the SuperSignal West Pico Chemiluminescence system (Pierce, IL, USA) and imaged with a Chemidoc XRS system (Bio-Rad, Hercules, CA, USA). The same membranes were probed with antitubulin and anti-GAPDH antibodies at a dilution of 1:1000. Densitometry analysis was performed using ImageJ software (NIH, National Institutes of Health, USA).

### 2.16 Statistical Analysis

The results are shown as the mean value of at least three independent replicates and their corresponding SD values, unless specified. Statistical significance was determined by the *t* test or one-way ANOVA and Dunnett’s multiple comparison test; values of *p* < 0.05 were considered statistically significant. Significance was indicated as follows: *p < 0.05, **p < 0.01, ***p < 0.001, and ****p < 0.0001. Statistical analysis of the data was performed using Prism-GraphPad software.

## 3 Results

### 3.1 Phenolic Profiling of the EVOO Extract

**Figure 1:**
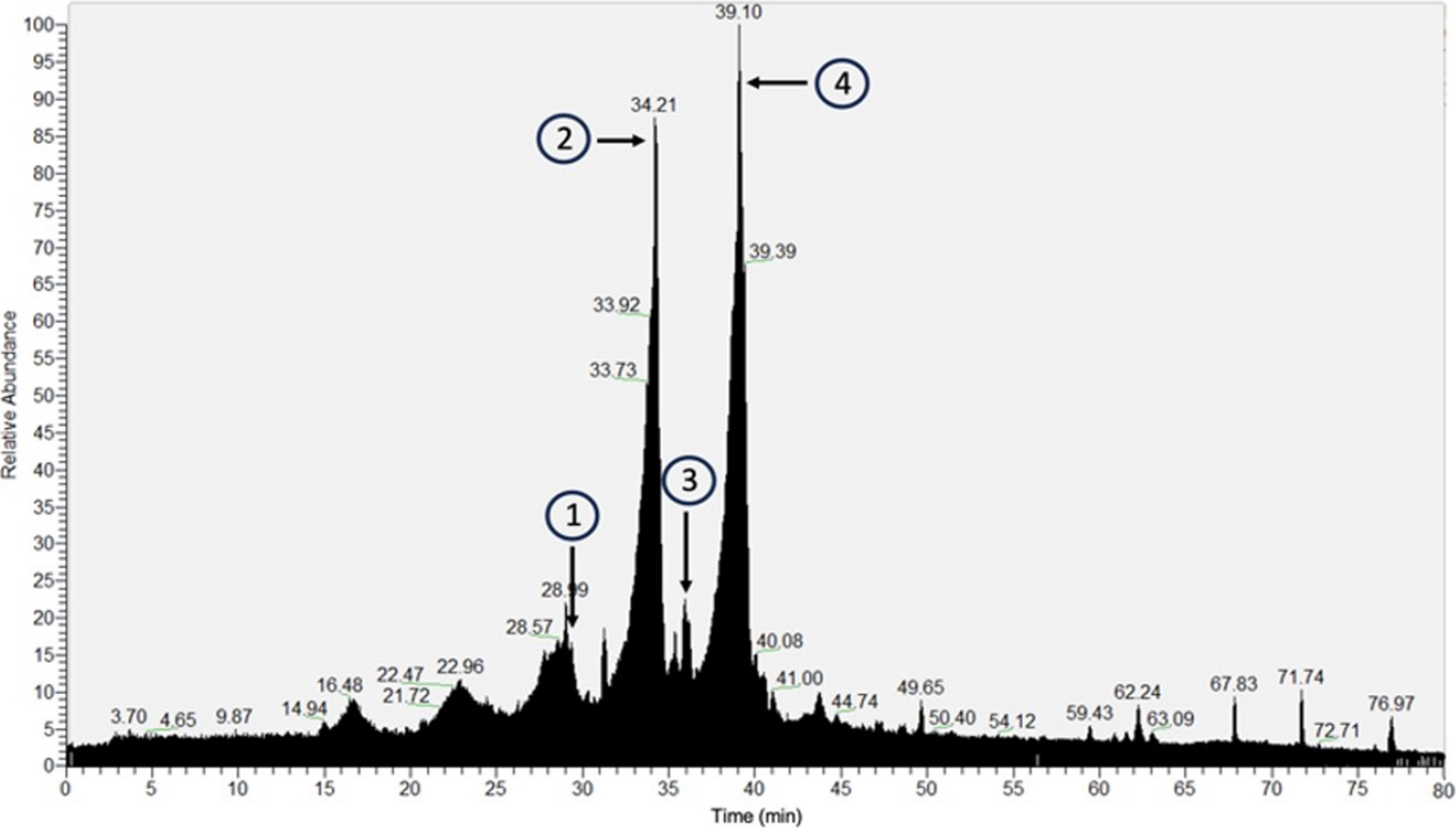
Phenolic profile of an extract from a “Picual” variety of EVOO. Total ion chromatogram for the EVOO phenolic extract acquired in full scan data-dependent acquisition MS2. The circles refer to the main compounds identified in the extract. The numbers correspond to 1: kaempferol; 2: oleuropein aglycon; 3: o-coumaric acid; 4: ligstroside-aglycone.

The obtained sample of the EVOO extract was analyzed to determine the phenolic composition. Overall, the most abundant molecules were kaempferol, oleuropein aglycon, o-coumaric acid, and ligstroside-aglycone. The total ion chromatogram (TIC) for the acquired sample is given in Figure 1, and the compounds identified in the EVOO extract are presented in Table 1. Mass spectrometry information related to the identification and confirmation of detected compounds is presented in the Supporting Information (Figure S1).

**Table 1:** List of Identified Compounds from the Extra Virgin Olive Oil Phenolic Extract

compound	molecular formula^a^	annotation MW^b^	*m*/*z*	calculated MW^c^	mass error (ppm)^d^	RT (min)^e^	reference ion
kaempferol	C15 H10 O6	286.04774	287.05493	286.04766	–0.29	29.371	[M + H] + 1
oleuropein aglycon	C19 H22 O8	378.13147	379.13898	378.13170	0.62	34.208	[M + H] + 1
O-coumaric acid	C9 H8 O3	164.04734	165.05441	164.04714	–1.27	36.004	[M + H] + 1
ligstroside-aglycone	C19 H22 O7	362.13655	363.14380	362.13652	–0.08	39.101	[M + H] + 1

a Molecular formula: assigned elemental composition.

b Annotation MW: theoretical molecular weight of assigned annotation; *m*/*z*: *m*/*z* value of the leftmost isotopic peak of the most common adduct ion for this compound.

c Calculated MW: neutral mass in Da retrieved from the measured leftmost isotopes of related compounds.

d Mass error (ppm): difference between the measured and theoretical molecular weight of assigned annotation in ppm.

e RT (min): retention time in min; reference ion: the most common adduct ion for this compound.

### 3.2 EVOO Extract Affects Endothelial Cells Survival at Relatively Low Doses

**Figure 2:**
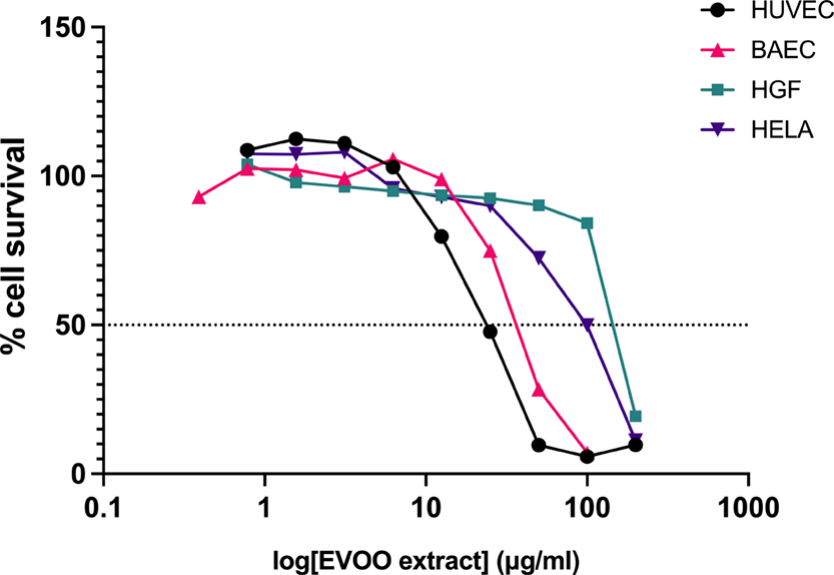
Survival of endothelial cells is affected upon treatment with EVOO extract. Survival curves of HUVEC, BAEC, HGF-1, and HeLa cells after 72 h of treatment with the EVOO extract. The dose at which cell survival reached 50% of the population represents the IC_50_ in each case. The calculated IC_50_ values were in μg/mL: 20 ± 4 in HUVEC, 43 ± 9 in BAEC, 148 ± 11 in HGF-1, and 107 ± 19 in HeLa. Data are shown as the media ± SD of at least three different experiments. Error bars for each cell line are shown in Figure S2.

The viability of two types of endothelial cells, HUVEC and BAEC in the presence of EVOO extract, was evaluated. Cells were submitted to a 72 h treatment regimen characterized by increasing doses of the extract. The resultant survival curves facilitated the determination of the IC_50_ value, representing the concentration at which 50% of the cellular population remains viable (Figure 2). Two non-epithelial cell lines were also analyzed, specifically the human gingival fibroblast HGF-1 and the tumoral HeLa cell lines. The calculated IC_50_, in μg/mL, were 20 ± 4 in HUVEC, 43 ± 9 in BAEC, 107 ± 19 in HeLa, and 148 ± 11 in HGF-1. This parameter was used as a valuable reference for optimizing dosage selection in the next assays.

### 3.3 Proteomic Analysis Unveils Altered Protein Levels Associated with Angiogenesis in HUVEC Following EVOO Extract Treatment

**Figure 3:**
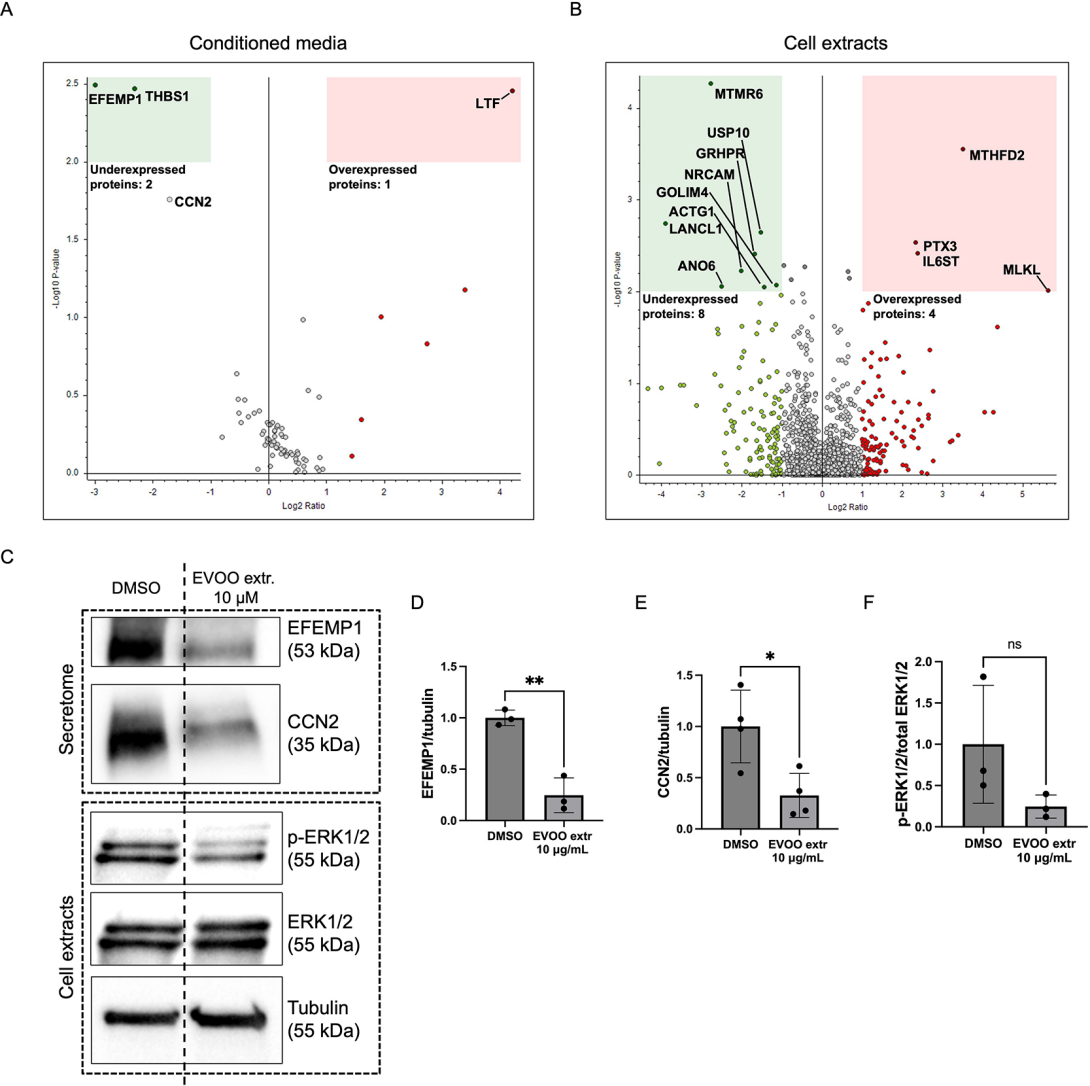
Differential protein expression in HUVEC treated with the EVOO extract. Volcano plots show deregulated proteins in conditioned media (A) or cell extracts (B) from HUVEC after treatment with the EVOO extract compared to the control condition. Proteins were considered upregulated (red square) or downregulated (green square) if fold-change values were >2 (Log_2_ Ratio >1, red circles) or <0.5 (Log_2_ Ratio <1, green circles), respectively. Among these proteins, changes in those with a ρ-value <0.01 were considered significant and grouped in red and green squares. Significantly deregulated proteins are labeled in each case. Gray circles, not significant. (C) Western-blot analysis of EFEMP1, CCN2, and ERK1/2 and its phosphorylated form was performed. Relative quantification (fold change) of the protein levels of EFEMP1 (D), CCN2 (E), and phosphorylated-ERK1/2 (F) was done. Means ± SD of a minimum of three independent experiments are shown, and Student’s *t* test was performed for the statistical analysis (*p < 0.05, **p < 0.01).

Proteomic analyses were conducted on conditioned media and cellular extracts obtained from HUVEC, treated with 10 μg/mL EVOO extract for 6 h. In the secretome, one protein, lactotransferrin (LTF), exhibited increased expression, while two proteins, specifically endothelial growth factor (EGF)-containing fibulin-like extracellular matrix protein 1 (EFEMP1) and thrombospondin-1 (THBS1), were decreased in the treatment group compared to the control condition (Figure 3A and Table S1 in Supporting Information). While not statistically significant according to the established threshold (p-value <0.01), CCN family member 2 (CCN2) was considered for further validation due to its comparatively low p-value compared to the other nonsignificant results. EFEMP1 is a matrix glycoprotein associated with decreased EGFR signaling and reduced cell adhesion and migration.^30^ Similarly, CCN2, also known as connective tissue growth factor (CTGF), plays a pivotal role in cell adhesion and growth.^31^ Interestingly, three proteins were exclusively detected in the conditioned medium of treated HUVEC (Table 1). Notably, interalpha-trypsin inhibitor heavy chain H2 (ITIH2), which exerts matrix protective activity through protease inhibitory action,^32^ was identified among these proteins.

Subsequently, cell extracts from HUVEC treated with the EVOO extract were analyzed, revealing 4 upregulated and 8 downregulated proteins when compared to the control condition (Figure 3B and Table S1 in Supporting Information). Among the upregulated proteins, mixed lineage kinase domain-like protein (MLKL) was identified, which is known to promote necroptosis.^33^ Additionally, pentraxin-related protein 3 (PTX3), a negative regulator of EGFR-induced angiogenesis,^34^ was observed. Furthermore, the downregulated neuronal cell adhesion molecule (NRCAM), which plays a critical role in cell adhesion,^35^ was detected. Remarkably, a subset of proteins was exclusively detected in either the control (Table 2) or the treatment condition (Table 3). Noteworthy, among the proteins not detected after treatment was dual specificity mitogen-activated protein kinase kinase 2 (MAP2K2), a member of the Ser/Thr protein kinase family known for its direct activation of the MAPK1/ERK2 signaling pathway.^36^ Additionally, epithelial membrane protein 1 (EMP1), which has been associated with enhanced cell migration,^37^ was absent following treatment. Finally, within the proteins exclusively present in HUVEC extracts after treatment, ran-binding protein 6 (RANBP6) stood out, recognized as an inhibitor of EGFR and STAT3 signaling pathways (Table 4).^38^

**Table 2:** Proteins Detected Exclusively in the Conditioned Media from HUVEC Treated with the EVOO Extract

gene symbol	accession^a^	description	coverage^b^ [%]	sum PEP score^c^
ITIH2	P19823	interalpha-trypsin inhibitor heavy chain H2 [OS = *Homo sapiens*]	3	9.849
CLEC3B	P05452	tetranectin [OS = *Homo sapiens*]	12	7.363
COMP	P49747	cartilage oligomeric matrix protein [OS = *Homo sapiens*]	4	7.409

a Accession: the unique identifier for the identified protein by the FASTA database used.

b Coverage: the percentage of the protein that is covered by the identified peptides.

c Sum PEP Score: a lower score indicates a lower probability of an incorrect match between the observed peptide spectrum (PSM).

**Table 3:** Proteins Detected Exclusively in the Control Condition of HUVEC Extracts

gene symbol	accession^a^	description	coverage^b^ [%]	sum PEP score^c^
MAP2K2	P36507	dual specificity mitogen-activated protein kinase kinase 2 [OS = *Homo sapiens*]	14	15.728
U2AF1	Q01081–2	isoform 2 of Splicing factor U2AF 35 kDa subunit [OS = *Homo sapiens*]	23	14.994
TOM1	O60784	target of Myb protein 1 [OS = *Homo sapiens*]	11	11.85
IVD	P26440	isovaleryl-CoA dehydrogenase, mitochondrial [OS = *Homo sapiens*]	8	8.802
PRKRA	O75569	interferon-inducible double-stranded RNA-dependent protein kinase activator A [OS = *Homo sapiens*]	8	6.883
EMP1	P54849	epithelial membrane protein 1 [OS = *Homo sapiens*]	45	6.395
TANC1	Q9C0D5	protein TANC1 [OS = *Homo sapiens*]	1	6.248

a Accession: the unique identifier for the identified protein by the FASTA database used.

b Coverage: the percentage of the protein that is covered by the identified peptides.

c Sum PEP Score: a lower score indicates a lower probability of an incorrect match between the observed peptide spectrum (PSM).

**Table 4:** Proteins Detected Exclusively in the Treatment Conditions of HUVEC Extracts

gene symbol	accession^a^	description	coverage^b^ [%]	sum PEP score^c^
POTEI	P0CG38	POTE ankyrin domain family member I [OS = *Homo sapiens*]	9	40.864
GTF2E1	P29083	general transcription factor IIE subunit 1 [OS = *Homo sapiens*]	11	9.383
ATG4B	Q9Y4P1	cysteine protease ATG4B [OS = *Homo sapiens*]	7	8.34
MRPL50	Q8N5N7	39S ribosomal protein L50, mitochondrial [OS = *Homo sapiens*]	15	6.99
RANBP6	O60518	ran-binding protein 6 [OS = *Homo sapiens*]	3	6.324
SMN1	Q16637-2	isoform SMN-delta5 of Survival motor neuron protein [OS = *Homo sapiens*]	24	4.338

a Accession: the unique identifier for the identified protein by the FASTA database used.

b Coverage: the percentage of the protein that is covered by the identified peptides.

c Sum PEP Score: a lower score indicates a lower probability of an incorrect match between the observed peptide spectrum (PSM).

To further validate our findings, two deregulated proteins, EFEMP1 and CCN2, were analyzed by Western blot. Additionally, the phosphorylation state of the ERK1/2 protein, directly activated by the deregulated protein MAP2K2, was analyzed. Notably, the results revealed a significant reduction in the levels of EFEMP1 and CCN2 in HUVEC treated under the previously mentioned conditions (Figure 3C–E). Furthermore, ERK1/2 phosphorylation, although noticeably reduced, did not show significant differences (Figure 3C,F).

These findings collectively suggest a complex interplay of protein expression changes in response to EVOO extract treatment in different human pathways, shedding light on potential regulatory mechanisms involved in angiogenesis and cell death. Thus, our subsequent steps involved a comprehensive in vitro characterization of this phenolic extract derived from EVOO, with the aim of assessing its potential as a modulator of angiogenesis and other related cellular processes. Because the following experiments were designed to evaluate general and likely conserved endothelial cell functions, a more practical and easily maintainable endothelial cell model, specifically the BAEC, was opted for.

### 3.4 Formation of Tube-like Structures by Endothelial Cells Is Abrogated upon Treatment with the EVOO Extract

**Figure 4:**
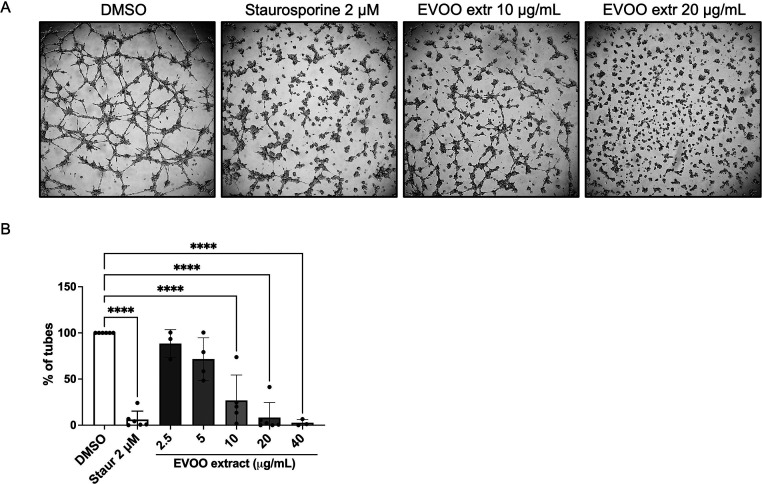
EVOO extract restrains the formation of tubule-like structures on Matrigel. (A) Representative images of endothelial tubular-like structures on Matrigel in the presence of DMSO, 2 μM staurosporine (negative and positive controls of inhibition, respectively), and different doses of the EVOO extract. 20 μg/mL corresponds to the MIC. Pictures were taken after 4–5 h of incubation. (B) Bar graph showing the number of sealed structures formed by endothelial cells. Data are shown as the percentage of structures normalized to the negative control. Means ± SD of a minimum of three independent experiments are shown, and one-way ANOVA + Dunnett’s multiple comparison tests were performed for the statistical analysis (****p < 0.0001).

The generation of tubular-like structures on Matrigel by endothelial cells is acknowledged as a representative model of the ultimate stage of angiogenesis, when cells rearrange to form a tubular structure that will eventually become the new blood vessel.^39^ Subsequently, to determine the potential of the EVOO extract to hinder the reorganization of endothelial cells into tubular-like structures in an in vitro setting, BAEC cells were cultured atop a Matrigel substrate while exposed to varying concentrations of the treatment. Notably, staurosporine was employed as a positive control in this experimental assay. Interestingly, 20 μg/mL of the EVOO extract was the lowest dose to completely abrogate tube formation, while 10 μg/mL partially inhibited the process yet still significantly (Figure 4). A dose response is observed in the graph, starting from 2.5 to 40 μg/mL.

### 3.5 EVOO Extract Interferes with the Migration of Endothelial Cells

Migration of endothelial cells through the extracellular matrix (ECM) is a key step in angiogenesis, where cells must relocate toward pro-angiogenic stimuli.^40^ Fibronectin, a major component of the ECM, plays an important role in the regulation of this process by activating cell-surface integrin receptors, following a cascade of intracellular signals that will stimulate actin and myosin in the cytoskeleton to promote movement of the cells.^41,42^

**Figure 5:**
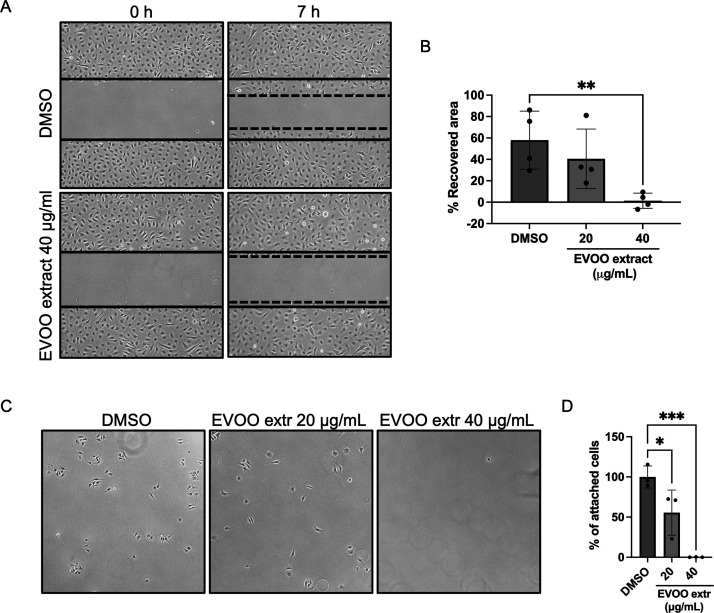
EVOO extract reduces the migration and adhesion of endothelial cells. (A) Representative images of the wound-healing assay performed in BAEC in the presence of DMSO (control condition) and 40 μg/mL of EVOO extract at time points 0 and 7 h. The solid lines represent the cell-free area at time 0 in each experimental condition, and the dashed lines indicate the area recovered by cells after 7 h. (B) Quantification of the area recovered by endothelial cells after a 7 h treatment with different doses of EVOO extract in the wound healing assay. Data are shown as percentages of the recovered area normalized by the control condition. (C) Representative pictures of the adhesion to fibronectin assay performed in BAEC in the presence of DMSO (control condition) and different doses of the EVOO extract, seeded on a fibronectin-covered surface. (D) Quantification of the percentage of cells that remained attached to fibronectin in the different conditions compared to the control. Means ± SD of three independent experiments are shown, and one-way ANOVA + Dunnett’s multiple comparison tests were performed for the statistical analysis (*p < 0.05; **p < 0.01; ***p < 0.001).

Subsequently, considering the prior findings regarding cell migration, we investigated the impact of the EVOO extract on cell adhesion to a fibronectin-coated substrate. Notably, at a concentration of 40 μg/mL, endothelial cell adhesion to fibronectin was completely inhibited, whereas a lower dosage of 20 μg/mL exhibited a partial inhibitory effect (Figure 5C,D).

### 3.6 Degradation of the Extracellular Matrix and Invasive Capacity Are Reduced in Endothelial Cells after EVOO Treatment

When angiogenesis is triggered, quiescent endothelial cells transite into an active state, leading to the degradation of the ECM to create space for migration.^40^ This process typically involves the secretion of proteases known as matrix metalloproteinases by these activated endothelial cells, which target specific components of the ECM.^43^

**Figure 6:**
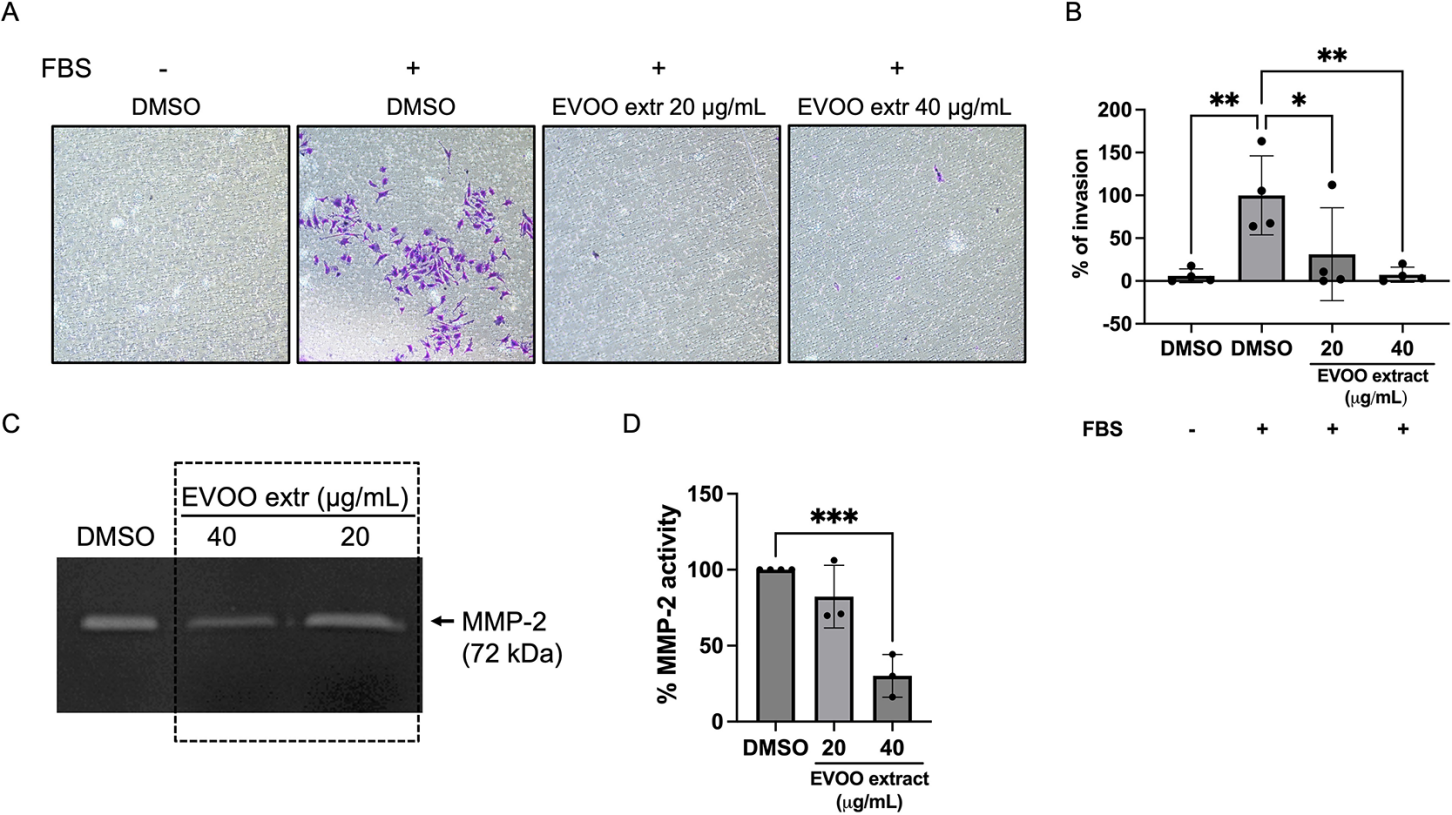
The invasive capacity of endothelial cells is abolished upon treatment with the EVOO extract. (A) Representative images of the invasion of BAEC through a Matrigel layer in the presence of DMSO or different doses of the EVOO extract. A negative control of invasion was added (left). (B) Quantification of the invasion as the percentage of endothelial cells that migrated to the bottom chamber. (C) Representative densitometry image of the gelatin zymography performed in conditioned media from BAEC, in the presence of DMSO or different concentrations of the EVOO extract. The bands correspond to MMP-2. Refer to Figure S3 for the unedited version of this zymography. (D) Densitometric quantification of the bands corresponding to MMP-2 as the percentage of MMP-2 relative activity. Means ± SD of a minimum of three independent experiments are shown, and one-way ANOVA + Dunnett’s multiple comparisons tests were performed for the statistical analysis (*p < 0.05; **p < 0.01; ***p < 0.001).

To investigate this phenomenon, we conducted an invasion assay using endothelial cells cultured on specialized transwells coated with Matrigel. These cells were then exposed to chemoattractant stimuli, enabling us to examine their invasive behavior. The results, depicted in Figure 6A,B, reveal that ECs effectively degraded the Matrigel layer and exhibited migration toward the stimuli in the control condition. However, this invasive capacity was significantly diminished post-treatment.

Furthermore, we utilized gelatin zymography to assess the levels of secreted matrix metalloproteinase-2 (MMP-2) in the conditioned media of BAEC under varying concentrations of the EVOO extract. Strikingly, the relative abundance of MMP-2 was significantly reduced upon exposure to 40 μg/mL of EVOO extract only (Figure 6C,D), suggesting that other elements, not only MMP-2, were affected in EVOO extract-treated BAEC, underlying the observed impaired invasion.

### 3.7 EVOO Extract Induces Apoptosis of Endothelial Cells in a Dose–Response Fashion

Indications that higher doses of the EVOO extract could potentially induce cell death in endothelial cells prompted us to carry out a more comprehensive investigation into this matter.

**Figure 7:**
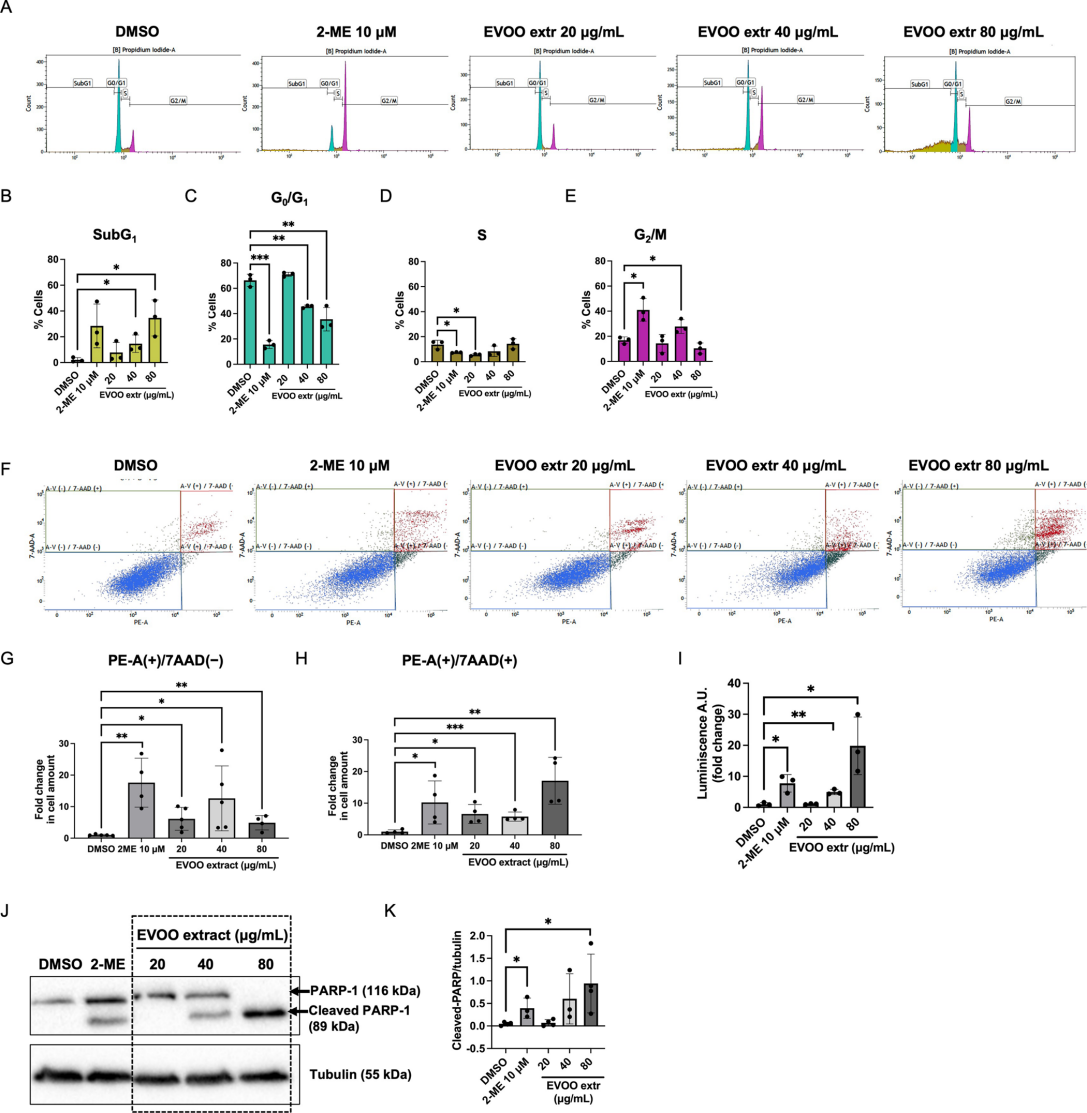
EVOO extract induces apoptosis in endothelial cells with a dose–response trend. A negative (DMSO) and a positive control of apoptosis (10 μM 2-ME) were included in all experiments. (A) Representative profiles of the cell cycle of BAEC in the presence of DMSO, 10 μM 2-ME, or increasing doses of EVOO extract. Quantification of cells in the (B) SubG_1_, (C) G_0_/G_1_, (D) S, and (E) G_2_/M phases of the cell cycle in either condition is shown. (F) Representative dot plots of cells positive or not for Annexin (PE-A) and/or 7-AAD staining and treated with DMSO, 10 μM 2-ME, or different doses of the EVOO extract. Quantification of cells in (G) early [PE-A(+)/7AAD(−)] and (H) late [PE-A(+)/7AAD(+)] apoptosis is included. (I) Relative activity of caspases 3/7 in BAEC in the presence of DMSO, 10 μM 2-ME, or different concentrations of the EVOO extract. (J) Representative blots of PARP-1 and cleaved PARP-1 in BAEC extracts in the presence of DMSO, 10 μM 2-ME, and different doses of the EVOO extract. Tubulin was used as an internal loading control. Refer to Figure S4 for the unedited version of this blot. (K) Densitometric quantification of the blots of PARP-1. Means ± SD of a minimum of three independent experiments are shown. Student’s *t* test was performed for the statistical analysis (*p < 0.05; **p < 0.01; ***p < 0.001).

The first step was to analyze the cell cycle distribution of endothelial cells in the presence or absence of the extract under study. The most noteworthy result was the progressive increase in the subG_1_ cell population that followed the dose increment (Figure 7A,B). These results were accompanied by a significant decrease in the G_0_/G_1_ phase when BAEC were treated with 40 and 80 μg/mL of the extract (Figure 7A,C). Additionally, a slight, significant decrease in the proportion of cells in the S phase was detected only with the dose of 20 μg/mL (Figure 7A,D), and a significant decrease in the G_2_/M phase was identified in BAEC treated with 40 μg/mL, compared to the control condition (Figure 7A,E).

Next, to validate a possible induction of apoptosis, we performed a series of more specific experiments. First, we studied the exposure of phosphatidylserine to cells with an Annexin V/7AAD kit. The results revealed that all doses of the EVOO extract significantly increased the population of cells that tested positive for Annexin V and negative for 7AAD [PE-A(+)/7AAD(−)], meaning early stage apoptosis (Figure 7F,G). Remarkably, the intermediate dose (40 μg/mL) caused the highest effect. Furthermore, the highest dose (80 μg/mL) exhibited the most pronounced enhancement in the proportion of cells positive for both staining types [PE-A(+)/7AAD(+)], indicative of late-stage apoptosis (Figure 7F,H). Thereafter, we studied the activity of caspases 3 and 7, which play a crucial role in the execution pathway of apoptosis, which is shared by both the intrinsic and extrinsic pathways.^44^ Interestingly, similar results were achieved when we checked the activity of caspases 3/7, where a clear increase in the activity was observed along with the dose rise (Figure 7I). Finally, the study of the cleaved levels of the poly(ADP-ribose) polymerase (PARP-1) protein by Western blot again disclosed a potential dose–response effect. Results showed no cleaved PARP-1 in cells treated with the lowest dose (20 μg/mL) of the EVOO extract, relatively similar amounts of cleaved and noncleaved PARP-1 after treatment with the intermediate dose (40 μg/mL), and only cleaved PARP-1 detected after the greatest dose (80 μg/mL) (Figure 7J,K). However, it is important to note that only 2-ME and the highest EVOO extract dose displayed statistically significant differences when compared to the control condition, potentially attributable to variability within this experimental context.

### 3.8 Alterations in the PI3K/AKT Signaling Pathway by the EVOO Extract

**Figure 8:**
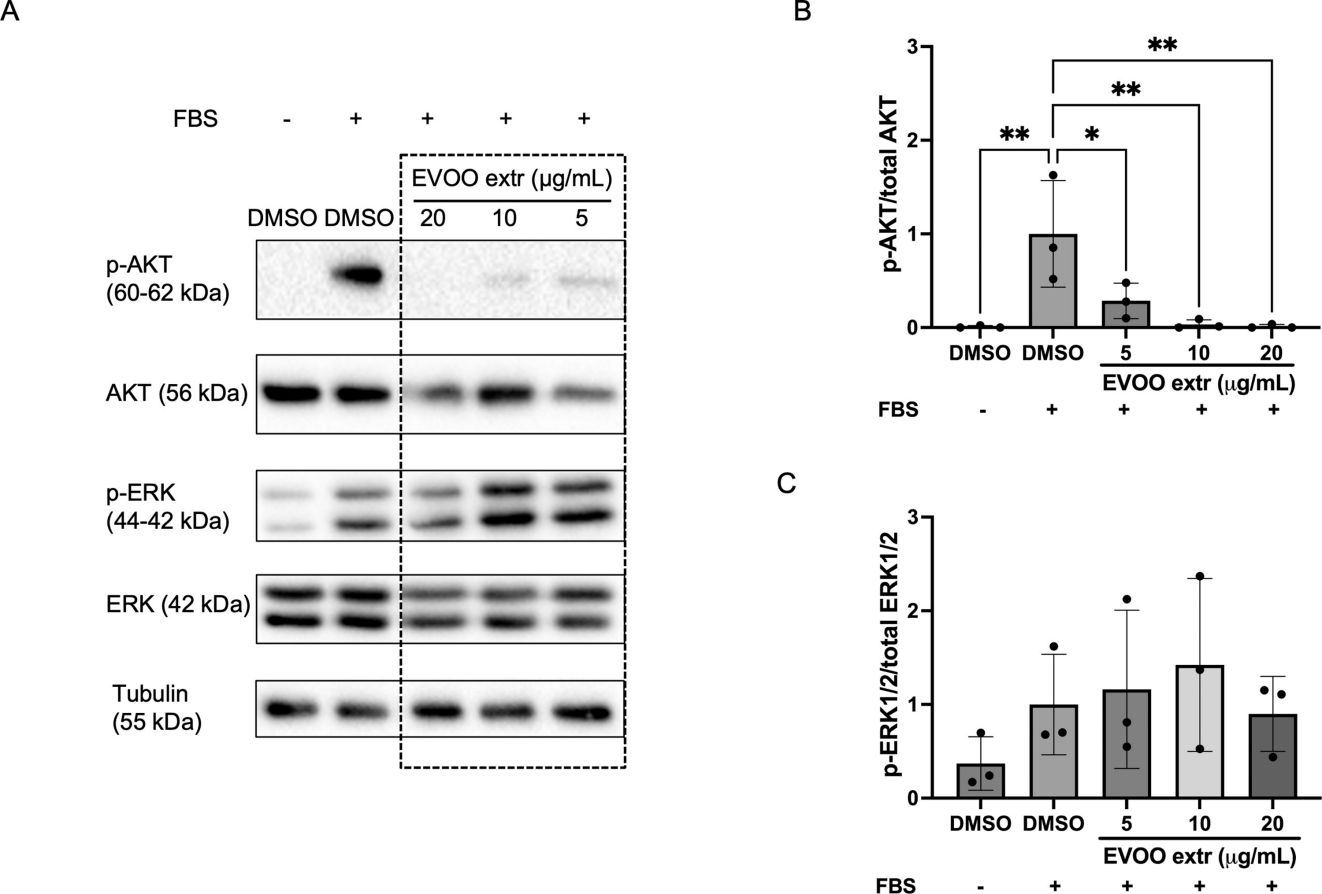
AKT phosphorylation is severely hampered in endothelial cells following EVOO treatment. (A) Representative blots of total AKT and ERK 1/2 proteins and their phosphorylated forms in BAEC extracts. Tubulin was used as an internal loading control. Similar results were obtained in, at least, three independent experiments. Refer to Figure S5 for the unedited version of this blot. (B, C) Densitometric quantification of the blots (fold change in ERK1/2 activation). Means ± SD of a minimum of three independent experiments are shown. One-way ANOVA plus Dunnett’s multiple comparison tests were performed for the statistical analysis (*p < 0.05; **p < 0.01).

Thus far, our findings have indicated significant impacts of the EVOO extract on the proliferation, migration, and viability of endothelial cells. Within this framework, the phosphatidylinositol 3-kinase (PI3K) and Akt/protein kinase B (PI3K/AKT) and the extracellular signal-regulated kinases 1 and 2 (ERK1/2) signaling pathways emerge as central regulators of the aforementioned cellular processes and actually wield a pivotal role over the regulation of angiogenesis.^45^ To illuminate this intricate dynamic, we conducted an exploration of the phosphorylation states of PI3K/AKT and ERK1/2 using Western-blot analysis. Notably, our results revealed a striking dampening effect of the EVOO extract on AKT phosphorylation in BAEC, even at doses as low as 5 μg/mL (Figure 8). Nevertheless, no discernible impact on ERK1/2 phosphorylation levels was detected by us. Remarkably, considerable variability among replicates was detected in the phosphorylation levels of ERK1/2.

## 4 Discussion

EVOO serves as a fundamental component of the health-related benefits associated with the Mediterranean diet,^8^ encompassing anti-inflammatory and antioxidant properties,^9−12^ as well as neuroprotective effects,^13−15^ and anticancer potential.^16−19^ Additionally, though to a lesser extent, it has been examined for its antiangiogenic effects.^20,21^

Within the molecules present in EVOO, phenolic compounds assume a pivotal position in driving its bioactive attributes.^3^ For this reason, studying the phenolic fraction of EVOO is intriguing as it allows for the acquisition of higher quantities of bioactive molecules compared to regular dietary intake, provided that the concentration of the various compounds in the mixture is safe. In this context, proteomic analysis is a powerful tool for unraveling the complex cellular responses when dealing with extracts containing a mixture of molecules such as phenolic compounds.

In this study, we examined the influence of an extract obtained from the “picual” variety of EVOO, originating from Jaén, Spain, on endothelial cell protein levels by UHPLC-MS. Our objective was to identify fluctuations that could potentially serve as indicators of effects on distinct cellular processes.

UHPLC-MS analysis of the phenolic profile of the EVOO identified four predominant molecules: kaempferol, oleuropein aglycon, o-coumaric acid, and ligstroside-aglycone. These compounds have been previously documented in EVOOs.^6,46,47^ Among these, oleuropein aglycon and ligstroside-aglycone were the most abundant, underscoring their significance. These compounds belong to the secoiridoid group, representing conjugated forms of hydroxytyrosol and tyrosol, which are highly concentrated in olive oil and extensively researched for their extensive health benefits.^48^ The group includes oleuropein and ligstroside aglycon isomers and the decarboxymethylated dialdehyde forms of oleuropein and ligstroside aglycons, better known as oleacein [(−)-oleacein; 3,4-DHPEA-EDA] and oleocanthal [(−)-oleocanthal; p-HPEA-EDA], respectively, which are also found at high levels in different EVOOs.^6^

First, we conducted some viability assays to check for the ideal dose with which to work and avoid cell toxicity. The results pointed to the mild toxicity of the EVOO extract in the endothelial cell lines HUVEC and BAEC, with an IC_50_ of 20 and 40 μg/mL, respectively.

Following this, a comprehensive proteomic analysis was conducted. In summary, the outcomes of the proteomic analysis revealed a deregulation of proteins involved in cell growth, adhesion, migration, invasion, and cell death processes. These findings will be explored in detail in a subsequent discussion. Outstandingly, all of these processes are narrowly related to angiogenesis.

Angiogenesis is the physiological process of vessel sprouting from pre-existent ones. During angiogenesis, endothelial cells switch from a quiescent to an active state to proliferate, migrate, and invade the ECM toward pro-angiogenic stimuli.^40^ This complex and tightly regulated mechanism plays a crucial role in various aspects of health and disease. In normal conditions, angiogenesis is involved in tissue growth and repair, such as during wound healing or development. However, when angiogenesis becomes dysregulated, it can contribute to the progression of numerous diseases, including cancer, as it enables the formation of blood vessels that supply nutrients and oxygen to tumors, facilitating their growth and metastasis.^49^ Therefore, understanding and controlling angiogenesis are significant focus in medical research, offering potential therapeutic effects.

In our proteomic study, we closely examined the conditioned media and cell extracts from HUVEC. Among the effects on the HUVEC secretome, our attention was drawn to the downregulation of the EFEMP1 and CCN2 proteins. Notably, previous research by Song and co-workers established that EFEMP1 plays a pivotal role in promoting angiogenesis and expediting cervical cancer growth in vivo.^50^ Intriguingly, cervical tumors marked by an overexpression of EFEMP1 exhibited elevated levels of VEGF and demonstrated increased microvascular density. Moreover, EFEMP1 protein facilitated the adhesion of Hela cells to HUVEC. Other studies have also linked EFEMP1 to tumorigenesis through the EGFR/EFEMP1 axis^30^ and the promotion of cancer cell migration by modulating MMP-2 and 9 via various signaling pathways [ERK1/2, nuclear factor kappa B (NF-κB)].^51,52^ Remarkably, silencing or knockdown of EFEMP1 in some of these studies resulted in reversion of an aggressive phenotype. Notably, Kwak and co-workers found that EFEMP1 knockdown attenuated hypoxia-induced breast cancer growth and metastasis.^52^ This protein has also been related to neovascularization in macular degeneration.^53^ These researchers also identified an upregulation of VEGF by EFEMP1 and observed increased tube formation and proliferation of HUVEC overexpressing EFEMP1. However, Song and colleagues did not detect these effects in HUVEC. Furthermore, plasma EFEMP1 has been also associated with brain aging and dementia.^54^

CCN2, also known as CTGF, assumes pivotal physiological functions in the development of essential tissues, such as cartilage and bone. This occurs through its direct interactions with ECM components, growth factors, and cell-surface receptors.^55^ An angiogenic role has been described for this molecule, not by directly stimulating a particular cell behavior, but as an organizer of microenvironmental cell society.^56^ Furthermore, CCN2 is deeply implicated in pathological alterations, including inflammation, fibrosis, and malignancies. A growing body of evidence underscores the role of CCN2 in promoting cancer initiation, progression, and metastasis through its regulation of cell proliferation, migration, invasion, drug resistance, epithelial–mesenchymal transition (EMT), and angiogenesis.^57^

Opposing the antiangiogenic evidence is the fact that THBS1, a potent antiangiogenic agent, is downregulated. This molecule inhibits endothelial migration and proliferation and induces endothelial apoptosis.^58^

ITHI2 was exclusively observed in HUVEC following treatment with the EVOO extract, strongly suggesting that its expression was induced by the treatment. This protein serves as a protease inhibitor, exerting tumor-suppressive functions that hinder metastasis. Notably, it has been consistently identified as downregulated in various cancer types.^32,59^

In our analysis of HUVEC extracts, we found the upregulation of MLKL and PTX3, along with the downregulation of NRCAM, which intrigued us. MLKL, traditionally recognized as the central regulator of necroptosis, has aroused discussions about its involvement in various cell death processes, including apoptosis and autophagy.^33^

PTX3, on the other hand, has demonstrated its capacity to inhibit fibroblast growth factors (FGF)-mediated angiogenesis and impede the growth and vascularization of FGF-dependent tumors, as observed in cases of fibrosarcoma.^34,60^

NRCAM, a cell adhesion molecule, has previously been identified in endothelial cells and has a known role in angiogenesis.^61,62^ Notably, it has also been implicated in tumorigenesis and cancer progression. In an in vitro model of gastric cancer, the silencing of the NRCAM gene led to the inhibition of cell growth, migration, and invasion while promoting apoptosis.^35^

Interestingly, MAP2K2, also known as MEK2, was not detected in HUVEC after EVOO treatment. As previously mentioned, MAP2K2 is a direct stimulator of the MAPK1/ERK2 signaling pathway,^36^ which has a central role in angiogenesis by promoting important processes such as cell proliferation and migration.^63^ Previous research has linked MAP2K2 to heightened proliferation and resistance to apoptosis in both endothelial^64^ and tumoral cells.^36,65^ Notably, it has been recognized as a central regulator in the intricate interplay between angiogenesis and inflammation.^64^

Conversely, the expression of RANBP6 appeared to be induced specifically by the EVOO treatment. Although the role of this nuclear importin in endothelial cells remains relatively unexplored, it has garnered attention as a negative EGFR regulator in cancer and has been found to be deleted or downregulated in certain cancer types.^38^

Provided the information extracted from our proteomic analysis, our following efforts went deeper into the characterization of the EVOO extract as a modulator of angiogenesis. Our approach involved a series of in vitro assays designed to simulate various stages of the process, done in BAEC.

The study revealed an inhibitory action of the EVOO extract in key angiogenic mechanisms. First, the extract disrupted tube formation at 10 μg/mL, showing promise for angiogenesis modulation. Furthermore, cell migration decreased significantly at 40 μg/mL, with the potential induction of cell death at 80 μg/mL, which will be further discussed. Moreover, reduced adhesion to fibronectin partially explained the decrease in migration, with both processes being critical during EMT. Furthermore, the extract notably reduced cell invasion, possibly linked to reduced MMP-2 release at 40 μg/mL, although other factors may contribute.

After that, our experiments focused on assessing the impact of EVOO on endothelial cell death. Cell cycle analysis evidenced an increasing proportion of cells in the SubG_1_ phase, and a decrease in the G_0_/G_1_ phase, indicating potential cell death induction. Additionally, flow cytometry results confirmed the induction of apoptosis, with different EVOO doses leading to varying stages of apoptosis. At a dose of 20 μg/mL, the proportions of cells in early and late apoptosis were similar, around 5%. However, the intermediate and high doses stood out with a high number of cells in early and late apoptosis, respectively, approximately 10% for the intermediate dose and 20% for the highest dose. These differences between doses are also supported by the arrest in G_2_/M caused exclusively by 40 μg/mL EVOO evidenced in the cell cycle study, prior to entrance to apoptosis. Moreover, caspase 3/7 activity and PARP-1 cleavage also demonstrated a dose–response pattern, further supporting the apoptosis-induction theory. Overall, our results suggest that the EVOO extract has the potential to induce apoptosis on endothelial cells.

Ultimately, the observed decrease in PI3K/AKT phosphorylation may explain the initiation of apoptosis at high doses. However, even at low doses when cell death is not evident, PI3K/AKT signaling remains significantly suppressed. This suggests the involvement of other downstream elements in the induction of apoptosis. The absence of inhibition in ERK1/2 phosphorylation evidences the fact that the effects on cell migration and proliferation are independent of this upstream signaling pathway, at least in BAEC. In HUVEC, despite the evidence hinting at decreased activation of the ERK1/2 signaling pathway (e.g., EFEMP1 under-expression and MAP2K2 disappearance following EVOO treatment, inhibition of migration, etc.), we were unable to establish statistical significance for this reduction. Nonetheless, discernible differences were observed in the blots, but it is likely that a high variability contributed to the lack of statistical significance. We believe that a more comprehensive investigation into the effects of this treatment on the ERK1/2 pathway is worthwhile and could provide further clarity on this matter. Interestingly, a recent article explored the significance of the mitogen-activated protein kinase (MAPK) pathway in cancer development and metastasis, with a focus on how dietary polyphenolic compounds can influence various MAPK subpathways to achieve anticancer effects, highlighting their potential for improving cancer treatment.^66^

Despite compelling evidence supporting the induction of apoptosis at the studied doses, it is worth mentioning that this does not impact our results concerning cell migration, invasion, or tube formation. These experiments had shorter incubation times compared to the cell cycle and apoptosis studies. Furthermore, some of these experiments demonstrated the effectiveness of the 20 μg/mL dose or even lower.

In summary, the phenolic compound mixture within the studied EVOO extract exhibits multifaceted antiangiogenic effects in endothelial cells, including reduced proliferation, migration, invasion, ECM degradation, and, at higher concentrations, the induction of apoptosis and other cell death mechanisms. These effects appear to be mediated through EGFR signaling, which is rendered less active due to decreased levels of EFEMP1 and CCN2 production and secretion, coupled with increased PTX3 levels and the induction of RANBP6 expression. Furthermore, the drastic reduction in AKT signaling observed in our in vitro experiments is noteworthy, given its pivotal role in cell proliferation and survival. Our in vitro findings align with reduced cell migration, which can be attributed to lower levels of EFEMP1, known to induce the ERK1/2 pathway associated with increased cell migration. Changes in the protein levels of CCN2 and NRCAM also support migration-related effects. Reduced cell adhesion is evident from our in vitro fibronectin adhesion assays and the decrease in NRCAM protein levels. The observed reduction in cell invasion and ECM protease production, as seen in our in vitro studies, is further substantiated by alterations in EFEMP1, CCN2, ITHR, and NRCAM protein levels. Additionally, the induced apoptosis detected in our in vitro studies is supported by reduced levels of AKT activation and changes in the protein levels of MLKL, NRCAM, and MAP2K2. Finally, the theory of angiogenesis modulation gains support from decreased levels of EFEMP1 and CCN2, both known inducers of VEGF signaling.

Among the phenolic compounds found in the examined EVOO extract, kaemferol and oleuropein aglycone have demonstrated anticancer and antiangiogenic effects by reducing VEGF expression and signaling through ERK1/2, AKT, HIF-1, and COX-2 in various cancer models, both in vivo and in vitro. Additionally, these compounds have been observed to decrease the levels of MMP-2 and MMP-9 expression and induce apoptosis. Furthermore, they inhibit angiogenesis-related processes like tube formation in endothelial cells.^67−72^ These findings corroborate our data for the EVOO phenolic extract containing these compounds. While ligstroside-aglycone and o-coumaric acid have received less extensive study, they have also shown anticancer activities.^16,73−75^ Additional phenolic compounds found in EVOO, such as hydroxytyrosol, have been extensively reviewed elsewhere due to their effects on cancer and angiogenesis, among other processes.^76−78^ Notably, our group recently published a study evaluating the antiangiogenic properties of the decarboxymethylated dialdehyde forms of oleuropein and ligstroside aglycons, known as (−)-oleacein and (−)-oleocanthal, respectively. This research demonstrates their ability to impede angiogenesis-related processes such as migration, invasion, and tube formation, along with decreased ERK1/2 and AKT signaling in endothelial cells.^27^

Our findings indicate that the phenolic extract from EVOO exerts clear antiangiogenic effects by modulating the expression of proteins in endothelial cells, leading to changes in proliferation, migration, invasion, adhesion, and survival. This is highly relevant in the context of dietary-based angioprevention.^79,80^ Indeed, there is substantial evidence indicating that we can benefit from EVOOs abundant in health-promoting phenolic compounds through regular dietary consumption, typically around 40 mL per day.^4,81^ Furthermore, similar EVOO-derived phenolic extracts are being evaluated in different clinical trials, especially concerning cardiovascular diseases and type-2 diabetes, reviewed in.^82^ In this line, the EVOO phenolic extract under study holds potential therapeutic applications in cancer and other angiogenesis-dependent diseases, such as macular degeneration or diabetic retinopathy. Equally important is the fact that the treatment can modify the proteins secreted by endothelial cells, expanding potential therapeutic effects to surrounding cells in the microenvironment. This naturally includes tumor cells, which closely interact with endothelial cells during tumor angiogenesis. In fact, the protein alterations induced by the EVOO extract evidenced by us clearly point toward antitumoral effects. Thus, the results of this study shed light on the novel bioactivities of phenolic compounds found in EVOO, highlighting their significance in the food and pharmaceutical sectors.

## Supporting Information Available

Additional data from the proteomic study, MS data from phenolic compound identification in EVOO, and individual survival curves of the different cell lines and original Western-blot images (PDF)

## Abbreviations Used

AGC: automatic gain control
BAEC: bovine aortic endothelial cells
CCN2: CCN family member 2
CTGF: connective tissue growth factor
DMSO: dimethyl sulfoxide
MAP2K2: dual specificity mitogen-activated protein kinase kinase 2
DMEM: Dulbecco’s modified Eagle's medium
EFEMP1: endothelial growth factor (EGF)-containing fibulin-like extracellular matrix protein 1
EBM-2: endothelial cell growth basal medium-2
EGM-2: endothelial cell growth medium-2 bulletkit
EMP1: epithelial membrane protein 1
EMT: epithelial–mesenchymal transition
EVOO: extra virgin olive oil
ECM: extracellular matrix
FDR: false discovery rate
FBS: fetal bovine serum
FGF: fibroblast growth factors
HCD: higher energy collisional dissociation
HGF-1: human gingival fibrobrasts-1
HUVEC: human umbilical vein endothelial cells
ITIH2: inter-alpha-trypsin inhibitor heavy chain H2
KIU: Kallikrein inhibitor units
LTF: lactotransferrin
HPLC-MS: liquid chromatography high-resolution mass spectrometry
MMP-2: matrix metalloproteinase-2
Meoh: methanol
MIC: minimum inhibitory concentrations
MLKL: mixed lineage kinase domain-like protein
NRCAM: neuronal cell adhesion molecule
NF-κB: nuclear factor kappa B
PTX3: pentraxin-related protein 3
PBS: phosphate-buffered saline
PARP-1: poly(ADP-ribose) polymerase
RANBP6: ran-binding protein 6
SD: standard deviation
MTT: thiazolyl blue tetrazolium bromide
THBS1: thrombospondin-1
TEAB: triethylammonium bicarbonate buffer
UHPLC: Ultrahigh-performance liquid chromatography
