# Supplementary material for: Antiangiogenic Potential of an Olive Oil Extract: Insights from a Proteomic Study
Source: J Agric Food Chem. 2024 May 29;72(23):13023–38. doi: 10.1021/acs.jafc.3c08851 (PMC11181319; doi:10.1021/acs.jafc.3c08851)
Supplement: Supplementary file 1 — jf3c08851_si_001.pdf [file jf3c08851_si_001.pdf]

Supporting Information

Antiangiogenic Potential of an Olive Oil Extract: Insights from a Proteomic Study

Ana Dácil Marrero[1,2,3], Casimiro Cárdenas[1,4], Laura Castilla[1,2], Juan Ortega-Vidal[5], Ana R.Quesada[1,2,3], Beatriz Martínez-Poveda[1,2,6]*, Miguel Ángel Medina[1,2,3]*

1 Departamento de Biología Molecular y Bioquímica, Facultad de Ciencias, Universidad de Málaga, Andalucía Tech, E-29071 Málaga, Spain; anadacil@uma.es (A.D.M.); quesada@uma.es (A.R.Q.); bmpoveda@uma.es (B.M.-P.); ccg@uma.es (C.C.)
2 Instituto de Investigación Biomédica y Plataforma en Nanomedicina-IBIMA Plataforma BIONAND (Biomedical Research Institute of Málaga), E-29071 Málaga, Spain
3 CIBER de Enfermedades Raras (CIBERER), Instituto de Salud Carlos III, E-28029 Madrid, Spain
4 Servicios Centrales de Apoyo a la Investigación (SCAI), Universidad de Málaa, E-29071 Málaga, Spain
5 Universidad de Jaén, Departamento de Química Inorgánica y Orgánica, Campus de Excelencia Internacional Agroalimentaria ceiA3, Jaén E- 23071, Spain
6 CIBER de Enfermedades Cardiovasculares (CIBERCV), Instituto de Salud Carlos III, E-28029 Madrid, Spain
* Correspondence: medina@uma.es, Tel.: +34-952137132; bmpoveda@uma.es, Tel.: +34-952131674

Table S1. Over and under-expressed proteins in the conditioned media of HUVEC treated with EVOO extract.

Gene Symbol	Accession	Description	Abundance Ratio (Treatment/ Control)	Abundance Ratio P-Value	Coverage [%]	Sum PEP Score
LTF	P02788	Lactotransferrin [OS=Homo sapiens]	18.538	0.003522289	3	7.149
EFEMP1	Q12805	EGF-containing fibulin-like extracellular matrix protein 1 [OS=Homo sapiens]	0.126	0.003222009	10	15.463
CCN2	P29279	CCN family member 2 [OS=Homo sapiens]	0.307	0.017503511	5	3.975
THBS1	P07996	Thrombospondin-1 [OS=Homo sapiens]	0.201	0.003409158	23	83.737

Table Keys
Accession: the unique identifier for the identified protein by the FASTA database used.
Abundance Ratio: displays the abundance ratios between the proteins in the selected samples.
Coverage: the percentage of the protein that is covered by the identified peptides.
Sum PEP Score: a higher score indicates a lower probability of an incorrect match between the observed peptide spectrum (PSM)

Table S2. Over and under-expressed proteins in HUVEC extracts after treatment with EVOO extract.

Gene Symbol	Accession	Description	Abundance Ratio (Treatment/ Control)	Abundance Ratio P-Value	Coverage [%]	Sum PEP Score
MLKL	Q8NB16	Mixed lineage kinase domain-like protein [OS=Homo sapiens]	49.789	0.009696756	13	11.651
MTHFD2	P13995	Bifunctional methylenetetrahydrofolate dehydrogenase/cyclohydrolase, mitochondrial [OS=Homo sapiens]	11.408	0.000282746	7	7.116
IL6ST	P40189	Interleukin-6 receptor subunit beta [OS=Homo sapiens]	5.233	0.003867634	3	5.155
PTX3	P26022	Pentraxin-related protein PTX3 [OS=Homo sapiens]	5.04	0.002946929	17	23.343
GOLIM4	O00461	Golgi integral membrane protein 4 [OS=Homo sapiens]	0.454	0.008571091	9	18.797
ACTG1	P63261	Actin, cytoplasmic 2 [OS=Homo sapiens]	0.367	0.008940958	90	376.665
USP10	Q14694	Ubiquitin carboxyl-terminal hydrolase 10 [OS=Homo sapiens]	0.349	0.002249886	9	15.451
GRHPR	Q9UBQ7	Glyoxylate reductase/hydroxypyruvate reductase [OS=Homo sapiens]	0.313	0.003896317	21	22.664
NRCAM	Q92823	Neuronal cell adhesion molecule [OS=Homo sapiens]	0.247	0.005977904	8	25.913
ANO6	Q4KMQ2	Anoctamin-6 [OS=Homo sapiens]	0.176	0.008768316	2	4.843
MTMR6	Q9Y217	Myotubularin-related protein 6 [OS=Homo sapiens]	0.146	5.40926E-05	3	4.861
LANCL1	O43813	Glutathione S-transferase LANCL1 [OS=Homo sapiens]	0.067	0.001824379	11	10.874

Table Keys
Accession: the unique identifier for the identified protein by the FASTA database used.
Abundance Ratio: displays the abundance ratios between the proteins in the selected samples.
Coverage: the percentage of the protein that is covered by the identified peptides.
Sum PEP Score: a higher score indicates a lower probability of an incorrect match between the observed peptide spectrum (PSM)

A

Name	Formula	Annot. ΔMass [ppm]	Calc. MW	m/z	RT [min]	Reference Ion
Oleuropein aglycon	C19 H22 O8	0.62	378.13170	379.13898	34.208	[M+H]+1

MS spectrum

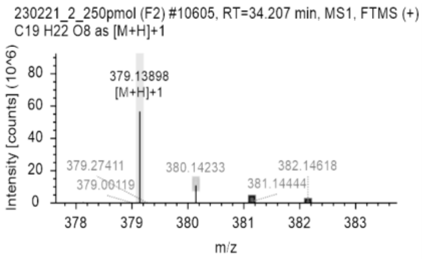

ddMS2 spectrum

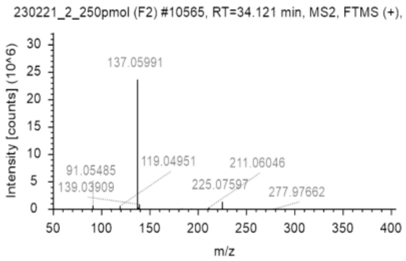

B

Name	Formula	Annot. ΔMass [ppm]	Calc. MW	m/z	RT [min]	Reference Ion
Ligstroside-aglycone	C19 H22 O7	-0.08	362.13652	363.14380	39.101	[M+H]+1

MS spectrum

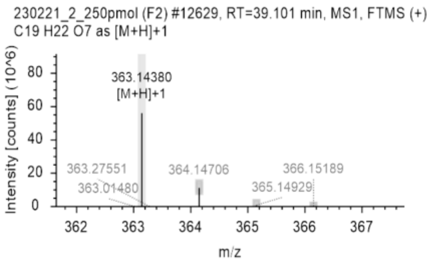

ddMS2 spectrum

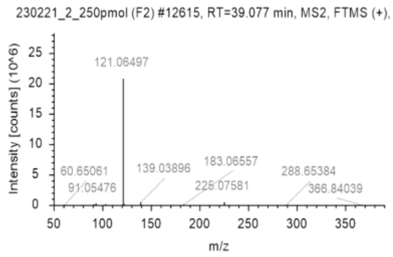

C

Name	Formula	Annot. ΔMass [ppm]	Calc. MW	m/z	RT [min]	Reference Ion
Kaempferol	C15 H10 O6	-0.29	286.04766	287.05493	29.371	[M+H]+1

MS spectrum

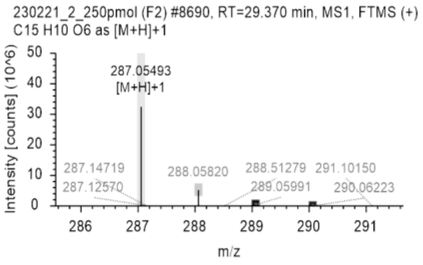

ddMS2 spectrum

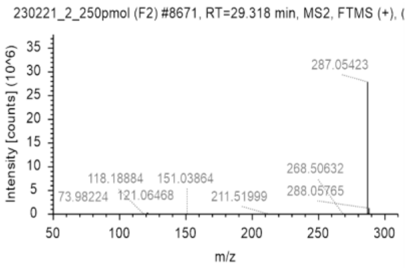

D

Name	Formula	Annot. ΔMass [ppm]	Calc. MW	m/z	RT [min]	Reference Ion
o-Coumaric acid	C9 H8 O3	-1.27	164.04714	165.05441	36.004	[M+H]+1

MS spectrum

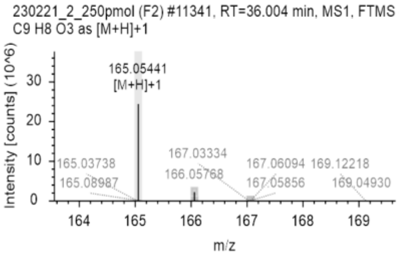

Figure S1. Detailed information from HPLC-MS related to the identification and confirmation of the detected compounds in the EVOO extract. Full MS and ddMS2 spectra (if applicable) of (A) oleuropein aglycon, (B) ligstroside-aglycone, (C) kaempferol, and (D) o-Coumaric acid are shown.

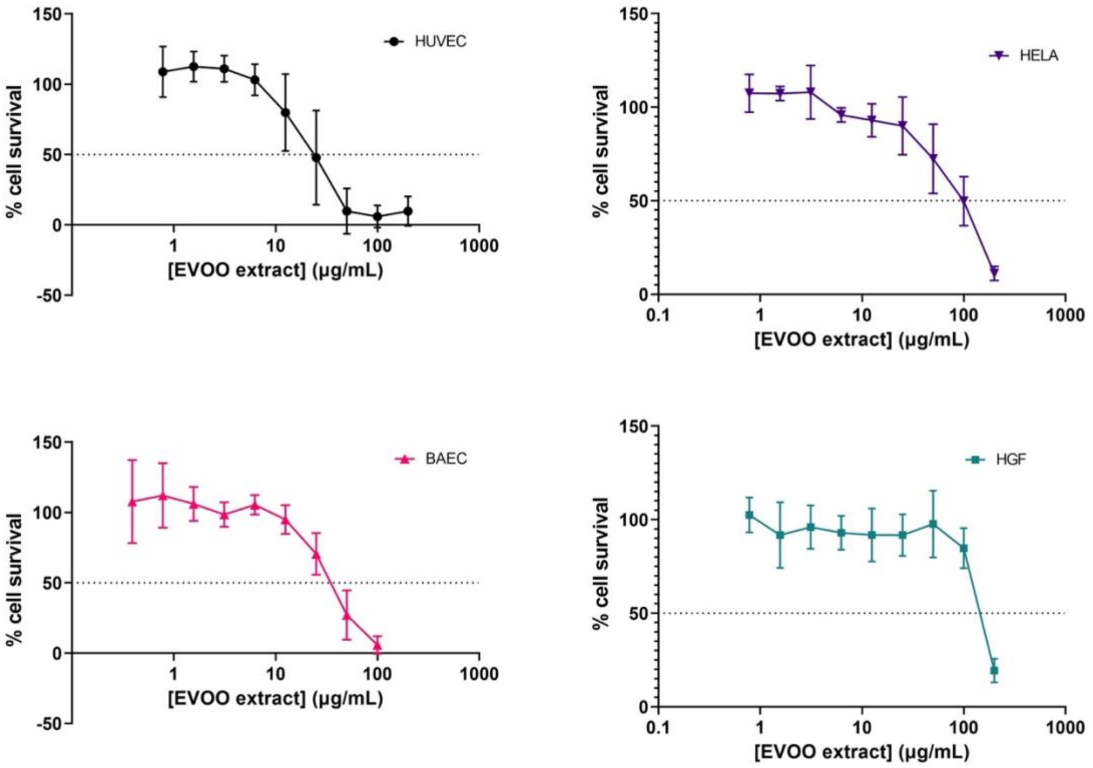

Figure S2. Survival curves of different cell lines (BAEC, HUVEC, HELA and HGF-1) in the presence of increasing doses of the EVOO extract. Data are shown as the media ± SD of at least three different experiments.

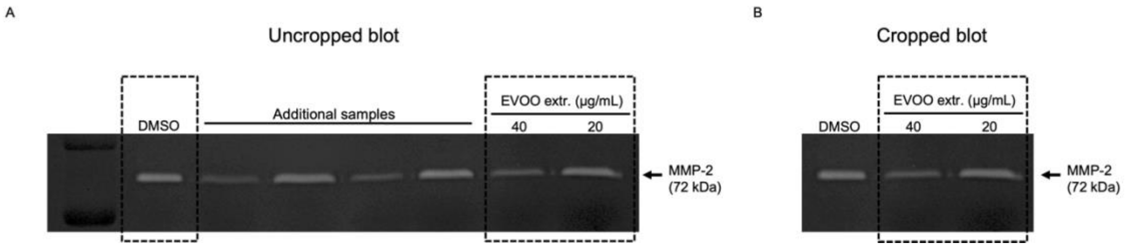

Figure S3. Zymography in Figure 6. Uncropped (A) and cropped (B) images of the zymography.

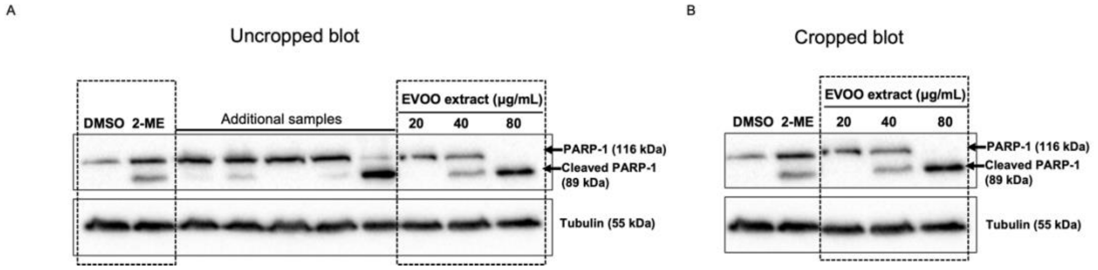

Figure S4. Western-blot in Figure 7. Uncropped (A) and cropped (B) images of the blot.

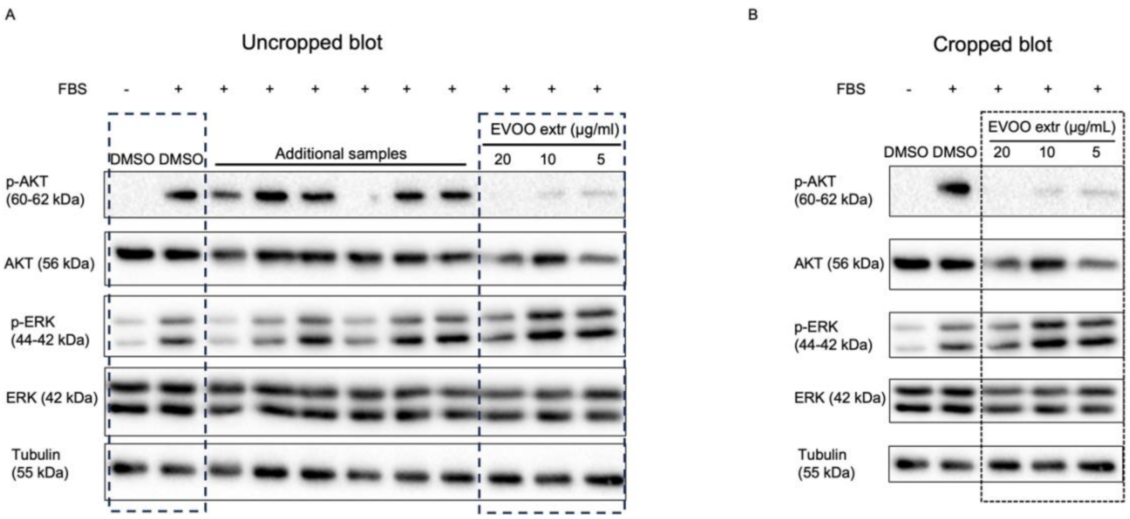

Figure S5. Western-blot in Figure 8. Uncropped (A) and cropped (B) images of the blot.
